# Care Coordination in Palliative Home Care: Who Plays the Key Role?

**DOI:** 10.5334/ijic.5466

**Published:** 2020-09-28

**Authors:** Emily Reeves, Brigitte Liebig, Reka Schweighoffer

**Affiliations:** 1 Fachhochschule Nordwestschweiz, University of Basel, CH

**Keywords:** palliative care, care coordination, home care, Switzerland, qualitative study

## Abstract

**Background:**

Clearly identified professionals who are appointed for care coordination are invaluable for ensuring efficient coordination of health care services. However, challenges to identifying roles in palliative care are well documented in literature. Notably, in order to meet high demands on palliative home care settings, many care practitioners perform tasks that surpass the responsibilities and regulations of their role, including care coordination. Without clearly defined roles, standards of care cannot be guaranteed. Yet, little is understood about who plays the key role in palliative home care.

**Aim:**

The present study aims to address the gap in the research by identifying who plays a key role in coordination in palliative home care.

**Methods:**

Interviews with general practitioners (GPs), nurses and relatives of palliative patients were carried out in Swiss cantons (Vaud, Ticino, Luzern and Basel) to identify key coordinators of care. Interviews were analyzed using content analysis and presented using grounded theory.

**Results:**

Findings indicated that there was considerable ambiguity of the key coordinator role. 1) Causal conditions of this phenomenon were; informality of professional roles and lack of communication between team members, 2) Consequences of this included; conflicting understandings of key coordinator role and family members feeling overburdened, 3) Strategies adopted by interviewees included; adapting or taking control of care coordination. These findings are highly indicative of areas for improvement for care coordination in palliative home care settings. Specifically, they underline a profound need for clear communication between palliative care service providers regarding which professionals assume a key coordative role, or who are delegated a coordinative role at any given time. Crucially, since the findings reveal that relatives are intimately involved in care coordination, the findings point to a lack of adequate financial and psycho-social support for relatives of palliative patients who are burdened with coordination tasks, without the appropriate recompense.

## Introduction

Clearly identified professionals who are appointed for care coordination are invaluable for ensuring efficient coordination of health care services [1,2]. Care coordination involves “the regulation of diverse elements into an integrated and harmonious operation” [3]. In palliative home care settings, individuals who play a key role in care coordination often include: general practitioners (GPs), nurses, advanced practice nurses and case managers [4]. These individuals are involved in coordination activities such as: outreach to patients by phone or mail; conducting face-to-face patient encounters; providing social support for patients; collecting, managing and exchanging patient data; supporting physicians; and backing up clinical and administrative staff [5].

Palliative home care teams allow patients to receive high-quality, personalized care in their own home setting [6]. The service is usually provided as a way of continuity of care from hospital to home, with the offer of up to 24-hour-a-day staff availability [7]. This is often only possible through supplementing additional members to a team or interchanging team members with shifting rotas, meaning that teams are commonly made up of changing staff members. Moreover, since home care requires intensive supervision and care, professionals must be readily available to respond to patient needs [8]. This can provide a challenging context for identifying those who play a key role in care coordination. Challenges to identifying roles in health care are well documented in literature [9,10,11] and are largely attributed to health care practitioners performing tasks that go well beyond the typical responsibilities and regulations of their role [11]. Notably, in everyday practice healthcare providers are increasingly performing roles that, in addition to clinical practice, concern organizational and managerial aspects, including care coordination [12,13]. Similarly, in order to meet increasing demands on health services, many care workers experiences “vertical substitution”, i.e. a role which is typically performed by a worker at a higher occupational station is now performed by a worker at a lower occupational station [14]. Crucially, these factors have been shown to contribute to adverse effects for care coordination as they can result in ambiguity concerning role recognition amongst care providers, i.e. “the situation where individuals do not have a clear direction about the expectations of their role” [9] and fuel role conflict, where individuals disagree about what the expectations are for a particular role [15]. Without clearly identified roles, standards of care cannot be granted [16] and the quality of coordination may be compromised [17]. Yet, little is understood about who plays a key role in care coordination. This is especially true for Swiss palliative home care, where research efforts have focused on specialized palliative care settings rather than home care in Switzerland [18]. Although some previous research in this field has identified GP’s as important actors in Swiss palliative home care [19], few studies have sought to address who the key coordinators are in palliative home care. The aim of the present study is, therefore, to address this gap in the research and thus explore who plays a key care coordinator role in palliative home care, from the perspectives of palliative home care teams.

## Methods

### Sample and context

Results are based on a qualitative study. The sample included 24 interviews with 12 general practitioners (GPs) and 12 nurses in primary palliative care including the home care setting, and 29 family members of palliative patients in different regions of Switzerland (see Table 1). Qualitative data was collected between January 2018 and April 2019. The relevant participants for the study were identified in each canton via internet searches and personal referral and were recruited via email invitation or telephone call. As Table 1 shows, the sample includes a larger number of women in the group of nurses and family members, while a slightly higher number of male GPs were interviewed. The mean age of those interviewees was 53 years of age for professionals and 59 years amongst family members.

**Table 1:** Demographic data of participants.

Participants	Number	Gender		Age (Mean)

		Female	Male	

GP’s	12	5	7	53
Nurses	12	10	2	53
Family members	29	20	9	59

### Ethical considerations

Formal research approval for this study was obtained from the Ethics Committee of Northwestern Switzerland (EKNZ) on the 29^th^ of August 2018 (Req-2018-00490). At the outset of interviews, participants received and completed an informed consent form for participation in the study and recording of interviews. Prior to interviews, explanations about objectives, reasons for recording interviews, voluntary participation, and confidentiality of data were given. The anonymity of participants was guaranteed to them and ensured through the exclusion of all personal identifiers from interviews.

### Interviews

Interviews were semi-structured and carried out in the language corresponding to the official language of three different language regions, i.e. German, French or Italian. Interviews lasted between 45–60 minutes and were conducted in a location determined by the participant (e.g. clinic, personal office, in a home setting or a public place). Interview guidelines were informed by concepts derived from a conceptual framework for the evaluation of integrated palliative care networks [20] and covered key topics to explore perceptions of coordination in primary palliative care. Interview questions for professionals included; “who in your opinion has a key role in your team when it comes to coordinating tasks?” and “how are tasks and responsibilities divided in your team?”. Questions directed at family members included: “who plays a key role in coordination in the palliative care team?” and “do you know how tasks and responsibilities are shared in the team?”.

### Data analysis

The first stage of analysis involved familiarization of the data, i.e. audio recordings and the transcripts. Audio files were either fully or partially transcribed in their original language by a professional transcriber or by one of the researchers, depending on the language of the interview. Two of the researchers are fluent in German, one is fluent in French and another is fluent in Italian. The transcriptions were translated where needed into the corresponding language of the researchers. The second stage was interpreting the data. The data from interviews was originally analyzed using ‘thematic analysis’ [21] to systematically identify and report content related patterns within the data. The first few transcripts were firstly coded manually and subsequent codes were grouped into themes of similar and interrelated concepts. A second researcher cross-checked to compare the codes that were applied to the initial transcripts and agreed on a set of codes to apply to all subsequent transcripts. This was done in order to ensure inter-coder reliability. Codes were agreed upon and were then clearly defined. The data in the subsequent transcripts were coded according to these themes. Lastly, remaining transcripts were organized and electronically coded, supported by qualitative data analysis software (MAX QDA) for efficiency. Later, the method of Corbin and Strauss [22], who espouse the development of ‘core categories’ (i.e. overarching concepts that tie the other categories together) was adopted. The paradigm scheme of Corbin and Strauss was used, which includes; the core phenomenon, causal conditions, context, consequences and strategies [22]. This was done in order to clearly illustrate the relationship between the categories found in the data.

## Results

**Figure 1:**
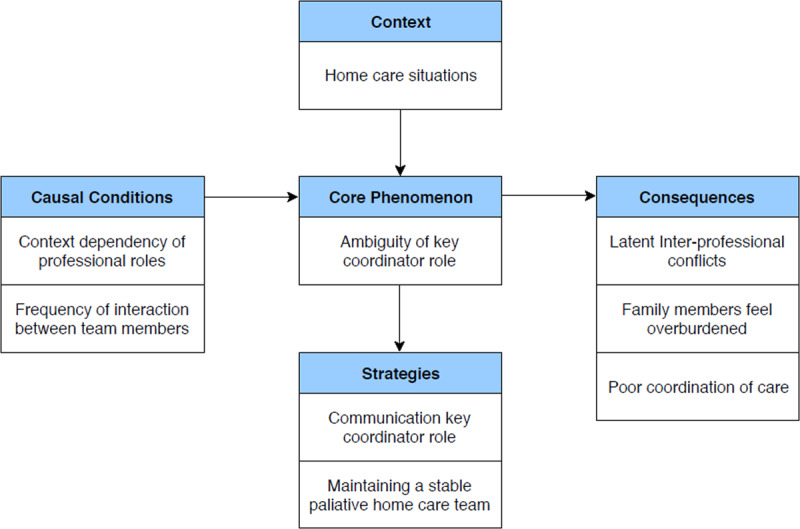
Ambiguity of the key coordinator role in palliative home care.

The aim of this study was to identify who plays a key role in coordination in a palliative home care setting, according to the perspectives of professionals and family members in the field. Findings indicated considerable ambiguity concerning who is responsible for care coordination in palliative home care contexts. Findings are presented using the conceptual framework proposed by Corbin and Strauss for the development of categories and subcategories [22]. The categories include; the identified core phenomenon (ambiguity of key coordinator role), causal conditions for this ambiguity of the key coordinator role, the context in which ambiguity of key coordinator role is found, the consequences of ambiguity of this key role and strategies adopted by the participants to mitigate this. The relationships between these categories are demonstrated in Figure 1.

### Core Phenomenon: Ambiguity of key role for coordination

The data revealed considerable ambiguity concerning the key coordinator role in the palliative home care setting. This was apparent in the discrepancy of responses in all three groups of interviewees regarding who is the key worker in primary palliative care teams. For example, whilst it was acknowledged by many participants that GPs are “theoretically” considered key coordinators, it was also argued that the key coordinator role does not fall so strongly on the GP in everyday practice: “In theory, yes, the GP is in charge – but in the end it is the nurse who is looking after all the care and making the decisions” (family member). This view is also supported by a professional in the field: “We see the doctor if we need him for a morphine prescription or something but I am looking out for everything on the ground here since I am with the patient night and day, almost every day” (nurse). Ambiguity is also evident in the lack of consensus regarding who obtains the key role in coordinating tasks. Notably, there are many cases of family members, nurses and GPs who all consider themselves to play a key role in coordination. Whilst a GP argues: “I am responsible for the care plan of patients and that they get the care that they need (GP), a nurse reasons the same: “I see to it that she (the patient) gets what she needs and when she needs it. If she needs something else, I can usually tell, and I can organize for her whatever it is that she needs” (nurse). Interestingly, family members also consider themselves as the key coordinators: “this is my job full time, it’s not a real job but this is what I do day and night. I am the one organizing things for my mum, every step of the way. I have everything here about my mother at my fingertips; I don’t need information from the nurses or anyone. I know exactly what medication she needs and when” (family member). When asked about who is the coordinator, a relative replies that they are as: “I know everyday life with my mum, it’s been years now. If she needs something else or something is wrong, I know what to do by now. I know how to tend to her because I learnt it all” (family member).

### Context: palliative care at home

Palliative home care is designed to provide symptom management while treating holistically (i.e. catering to physical and psycho-social needs) and allowing patients the choice of receiving high-quality, personalized care in their private home setting. The service is often provided as a way of continuity of care from hospital to home, with the offer of up to 24-hour-a-day staff availability. This is commonly only feasible by adopting additional members to a team who have complimentary working hours, meaning that teams are often made up of changing staff members. Moreover, since those in home care require intensive supervision and care, professionals must be readily available to respond to patient needs and changing states of health. This can provide a challenging context for identifying clear coordinators across situations. Some nurses report that care teams frequently change members which often results in ambiguity concerning who is responsible for care coordination or decision making: “It really depends who is around because it’s not always the same people working (…) if somebody new comes in (to the team) and you have to teach them from scratch what to do (…) they don’t really know how we do things or who they should go to in situations” (nurse). This perspective is shared by family members who express the belief that new members to the team can cause confusion because they don’t adhere to the implicit norms that allow for smooth coordination: “normally, people know they should contact Maria (nurse) if there is anything, but if the person is new (to the team), then they don’t necessarily get it straight away” (family member).

### Causal conditions for the ambiguity of key coordination roles

The findings indicate two key factors that contribute to the ambiguity of the key role for care coordination in palliative home care settings, including; difficulty of formalized roles and a lack of communication between team members in primary palliative care.

#### Context dependency of professional roles

Findings indicate that interviewees experience their roles depending on specific conditions and situations. A ‘formalized’ role, in the sense of standardized, normed behavior is difficult to achieve in the palliative care setting at home. Interviewees described their understandings of their roles in the team to be a result of circumstance. For example, the majority of nurses who considered themselves to have key roles in coordinating tasks felt this way as they are most often available for and in contact with patients: “of course we are aware of everything to do with their care plan because we see them (the patients) every day” (nurse). Moreover, some nurses and family members propose that the GP plays a key role in coordinating tasks in the case of critical situations since they are often not as visible as nurses: “I see the nurses almost every day. On the other hand, I see the GP when there’s bad news.. in the case that my mum really needs something” (family member). However, it is also reported that the GP is not always available in a critical situation. Therefore it is the nurse who makes the decisions in the moment: as one mother describes: “my daughter was having a terrible episode, she needed an ambulance but the doctor took two hours to arrive to us, so in the meantime, it was the nurse who did everything for us” (family member).

#### Frequency of interaction

Although many participants report that the GP is the ideal care coordinator, nurses and family members highlight that they have limited contact with GPs. The frequency of contact between actors determines to whom central roles are assigned in in the home care setting: “Sure, maybe I should ask the doctor but it’s easier for me to ask the nurse because I see her more often” (family member). Most nurses and family members agreed that they did not have much contact with GP’s compared to nurses. As one nurse states: “I work daily with other nurses and I don’t often see the doctor” (nurse). However, there are some exceptions as individuals living in rural areas report higher visibility of GP’s: “I am lucky because my family doctor lives in this little village. Here, I can even walk to see him if I need to” (family member). However, if GPs are not readily available, the assignment of coordination roles is dependent upon situational requirements, independent from professional roles, as the following quotation shows: “maybe we ring the doctor but he’s not available and then what are we supposed to do, just wait? No, we ask the nurse for the next steps, we can’t wait too long for things or my mother suffers” (family member).

### Consequences

#### Latent inter-professional conflicts

Findings reveal contradicting beliefs regarding who has the key role in coordinating tasks in palliative home care settings. For instance, while one GP describes that: “I am who has the final say of course, although I delegate tasks to the nurses” (GP), some nurses perceive the GPs role to be far less significant: “at the end of the day, we need them to sign off prescriptions” (nurse). The ambiguity of key coordinator roles also causes the relationships within the care team to suffer and be charged by latent inter-professional conflicts. Since the central task of coordination, if not clearly defined, is often mixed with a leadership function. Notably, in the case of GPs, the lack of frequent interaction with patients in the home care setting can result in information gaps, which in turn hinders decisions from being made, as the following quote illustrates: “I have to fill the doctor in and it’s embarrassing because I respect his rank but I have to find a way to tell him what needs to be done” (nurse). As a head nurse describes, the role that a professional in the team takes is often determined by who appears to be present at the time that coordination of care must take place: “Yes, theoretically, the GP is usually the one who ought to have the say in what should happen to the patient.. but the reality is that he’s not always around and then it falls on me to make choices for the patients care” (head nurse).

#### Family members feel overburdened

Many family members report feeling highly responsible for the care coordination of their family member: “we take on decisions for them, and of course they (palliative patient) want to retain their autonomy as long as possible but we have the responsibility” (family member). Moreover, since many family members have been taught how to administer treatment and feel that they know their relative best, they consider themselves to be key players for care coordination: “Since I am the one who is with them at all times, I have learned what they need and when they need it” (family member). Further, family members report that frequent contact with the PPC team makes them feel vital to the care process “I have a lot of contact with the nurses and I attend visits to the doctor, so I am heavily involved” (family member). In one case, the family member reports being crucial for decision making since the medical condition of the palliative patient is such that they are unable to make informed decisions for themselves: “I cannot avoid being involved in this process. My daughter cannot think for herself, so I am obligated to be involved in every step of her care plan and I make the decisions for her” (family member). At the same time, many family members report feeling overwhelmed in their role: “I gave up my job a long time ago and it (care for family member) took over my life, I tried to keep my head above water but it so hard sometimes, I would just burst into tears thinking I couldn’t keep at this for much longer” (family member). The following quotation shows in more general terms the extraordinary burden that arises from care situations in which family members assume a central role: “I work more now than I ever did in my entire life.. and that is saying something! I can’t leave the house for a coffee with a friend because I know at 4pm I have to give my daughter her treatment”.

### Strategies

The interviews illustrate several strategies to cope with the ambiguity of coordinating roles. Critically, the clear communication of the coordinative role and maintaining stable palliative home care teams appear to mitigate ambiguity of the coordinator role.

#### Communication of coordinator role

Respondents discuss the importance of team members communicating their roles frequently and unambiguously to others, to avoid confusion and to facilitate the coordination of tasks. One of the primary reasons for this seems to be that the situations of patients can be quickly changing and thus require immediate action. As one participant acknowledges: “I also cannot be around 100% of the time, so this is something I have to accept and I leave it to the head nurse to decide on things I would usually have a say on” (GP). In response to this, professionals describe that they clearly communicate when they are in a coordinative role: “I tell family members to contact me if they have any questions or doubts in their minds about anything. They always seem to respond well to this and I think it solves a lot of issues because they don’t need to wonder who to contact in case of something; they know I’m here” (head nurse). Moreover, if a professional delegates the coordination role to another professional, this is clearly communicated to other members of the palliative team. One GP explains: “When I know I won’t be around, I tell the others to contact the head nurse for everything to do with the patient. Of course, some things only I can be responsible for, like prescriptions, but on the whole, the head nurse can be left in charge” (GP). One family member also describes how when new members join a team they explain to them who is usually responsible for the care coordination of the patient: “Since they’re new they don’t always know the nurse so I sometimes have explained to new members that this particular nurse is the person to go to in case of anything or see this other person if you don’t know how about timings of things!”

#### Maintaining team stability

Professionals and family members equally experience the confusion in key coordination roles resulting from frequent changes in the team. In this situation it seems important to most of the interviewees to keep the team as stable as possible. The familiarity with other members of the team is identified as highly relevant to inform team members of professional roles – roles of coordination especially – since working in a team for a long time creates routines and expectations with respect to inter-professional interaction. As a nurse explains: “Since I have been working with the same team every day, I don’t doubt that I have to ask the other nurse about the schedule, I know that she is the person responsible for that” (nurse). Moreover, the implicit character of coordination facilitates everyday interaction: “you don’t need to ask who is doing what, you’re already well informed” (GP). In order to maintain this stability, some describe trying to schedule their working hours with the same team members they’re used to working with, as one nurse describes: “It’s not always possible, but Elizabetta (nurse colleague) and I try to be on the same shifts together when we can, especially when it’s the same patient we know well. Then we try to both attend them or be working with them at the same time”. Family members in particular report that having worked with the same members of a team aids in understanding who is the key coordinator: “Since I know everyone really well, we have our routine going and that means I know who is in charge of what and when!” (family member). In order to further facilitate this, one family member reports specifically requesting a nurse to treat their family member: “I request that Maria comes, when I know that things are intense with my mum and that she might need more help because I feel better that way than if it’s just someone else who shows up. Usually she’s available for us!”

## Discussion

Ambiguity of key coordinator roles is a widespread phenomenon in palliative home care settings in Switzerland. This is one of the most important results of this explorative study, which also indicates that the ambiguity is predominantly caused by the context dependency of roles and a lack of communication between palliative care team members. The negative consequences of ambiguity concerning the key coordinator role are manifold and include; conflicts in inter-professional collaboration and family members feeling over-burdened with care coordination. On the other hand, results indicate that clear communication of situated key roles, as well as the maintenance of stable palliative care teams can facilitate care coordination in palliative home care settings.

The ambiguity concerning who plays a key role in care coordination is somewhat surprising, given the strong emphasis placed on designated care coordinators in palliative care settings [22,23]. Yet, the results overlap meaningfully with other empirical findings, which demonstrate that role ambiguity in health care settings can often arise out of the demanding nature of care, encouraging professionals to take on roles spontaneously [22] despite the demands of the role going beyond what might typically be expected [24,25]. In line with this, our findings indicate that since the palliative home care team often changes to meet patient needs and cater to full-time care rotas, the coordinator role is mostly determined in the context of specific conditions, if not in the individual case [20]. For instance, nurses reportedly felt they played a key role in care coordination when GP’s were not present, reflecting the context-dependency of the coordinator role in this specific setting. This also builds on the growing body of research that raises awareness that healthcare providers are performing roles that, in addition to clinical practice, concern organizational and managerial aspects [12,13]. Furthermore, it supports empirical findings that demonstrate that nurses shift from dependence on physicians to a new, more responsible and autonomous role [26]. In addition, a lack of communication between care providers adds further confusion and disclarity to understanding roles. For example, whilst responses from GPs, nurses and family members indicate that GP’s ideally play a key role for care coordination, it is apparent that a lack of communication diminishes the perceived significance of their role in this regard. This is somewhat to be expected given that contact with others is important for establishing roles [27,28]. However, the findings also indicated that if professionals communicate clearly when they are in a coordinative role, this could be sufficient to mitigate ambiguity about who is the key coordinator in a given circumstance. This principle is already effectively adopted in trans-professional teams, where member roles are flexible rather than fixed [23]. Crucially, the effective functioning of such teams relies heavily on team members communicating their roles clearly [29], something that the results from this study can only attest to, given the negative consequences associated with the poor communication of roles. Whilst clarifying roles may mitigate issues with identifying key care coordinators, it could be argued that these issues could potentially be easily resolved by hiring a designated care coordinator, i.e. and individual who is clearly appointed for the job of coordination of patient care. This would make sense, given that the results essentially allude to the drawbacks of care coordination being carried out informally by health care professionals, who don’t necessarily have the specialized training, nor the time, for coordinating care as official care coordinators do. Firstly, clearly identifying one designated professional could, quite basically, answer the question of who is responsible for coordinating a task, independent of the context or situation. In addition, since care coordinators are soley preoccupied with coordination tasks and do not have to invest their time for other patient demands, as other health care professionals do, they can be fully dedicated to the role, without other commitments. Moreover, the data suggests that since health care professionals take on care coordination tasks, in addition to their already busy agendas catering to patients, a care coordinator could be beneficial for relieving some of the extra workload and pressure surrounding care coordination. Unsuprisingly, many countries have sought to implement care coordinators in palliative care teams to address these considerations and reaped the benefits with respect to coordination and quality of care, as is well documented in literature [22,23]. Different health care systems may of course adopt care coordinators to varying degrees and capacities, depending on the demands from the care services. In the United Kingdom (UK), the United States (US) and Canada, care coordinators are a long standing, fundamental pillar of palliative care services [30]. However, whilst adopting official coordinators into palliative care services is a crucial development, it is still not standard practice in Switzerland. Initiatives to encourage the implementation of advance practice nurses and case managers in Swiss palliative care services are only recently gaining attention in the field of palliative care [31]. However, as the data suggests; Switzerland is still behind in this regard, since care coordination is seemingly adopted informally by health care professionals, rather than by an official care coordinator. Moreover, as already discussed, Swiss cantons implement practices independently from one another, which means that there is no standardized approach to how the coordination of services is managed in Swiss palliative care.

### Implications of role ambiguity

Effective role recognition is considered an important element of high-quality care coordination [20]. Conversely, as previously demonstrated in health care settings [32,33] and echoed by the present findings, role ambiguity contributes to conflicts in role understanding and poor coordination of care in home care settings. Importantly, the data reveal the negative impact that this has on family members of palliative patients. Essentially, the lack of clarity with respect to care coordinator roles contributed to family members feeling responsible for and overburdened with care coordination. This finding is in line with prior research that demonstrates that family members of palliative patients feel highly involved in the care of their loved ones [34]. It is reasonable to assume that families of palliative patients desire a specific role in the home care situation for several reasons; notably, the private character of the care at home, assigns additional responsibility to family members, which may only partially be shared by ‘visiting’ professions [34]. Moreover, palliative education is increasingly accessible for family members, encouraging their involvement in care coordination and its related tasks [31]. This reflects that care coordination demands the appropriate formation and competencies [2]. Importantly, it is a role that requires the appropriate recognition and support [5]. Yet, whilst responses from professionals imply that they see either their own role or that of another professional as significant for care coordination, they did not share the same belief about the involvement of family members in coordination. This might allude to the role of family members either being overestimated by the relatives themselves or underestimated by the professionals in the study. Critically, if it is the case that family members play a key role in coordination but that professionals fail to recognize it, it could mean that family members are not receiving the emotional or practical support they may require for this role. Unfortunately, the evidence suggests that family members of palliative patients in Switzerland are already under-supported. Whilst there is financial support available for family carers in some other European countries, the UK and the US [35], Switzerland does not yet have regulations which allows for this [36]. This in itself may contribute to the experiences of the burden that many family members report in the data, since they must finance themselves whilst caring for their relative. This is not only costly but particularly challenging, if not impossible, for individuals who are no longer in employment because of their obligations to their family member, as is the case for many [37]. Adequate reimbursement in health care is of upmost importance for ensuring good quality care and alleviating stress for carers [29,34,36]. Beyond that, appropriate training and emotional support should be readily available to them in order to match their involvement in care coordination. Finally, in order to diminish role ambiguity in palliative home care teams, team members should experience similar training; this would also serve to consolidate team understanding and functioning of care coordination roles.

## Conclusions

This study reveals that there exists considerable ambiguity concerning who plays a key role in care coordination amongst professionals and family members of palliative patients in palliative home care settings. Moreover, the findings reveal that this ambiguity can be attributed to the fact that the key coordinator role is often context dependent, rather than a fixed position. Moreover, the findings affirm previous studies, which highlight that poorly defined professional roles contribute to poor coordination between professionals and poor quality of care. Importantly, the results shed light on the negative impact that ambiguity concerning key coordinators has on family members, who consequently take on care coordination roles themselves and are over-burdened and financially under-supported. The results ultimately stress the importance of clearly communicating key roles to all members of the palliative teams, including relatives, as well as highlighting the need for adequately supporting those who are in coordinating positions. The outcomes of this study are of interest to health care practitioners, policymakers and researchers involved in the support of care coordination in palliative home care settings. Crucially, future initiatives to encourage the effective communication of roles and to consolidate standardized palliative care team understandings regarding care coordination roles ought to be invested in. Likewise, ensuring that family members of palliative patients are ensured the appropriate education, psycho- social and fincial support that reflects their involvement in care coordination warrants special attention.

## Limitations

The explorative design of this study does not allow for generalizing the experiences, attitudes and beliefs found here with regard to who plays a key role in care coordination in palliative home care settings. Further, the results are based on the findings from interviews in only four Swiss cantons. Whilst the cantons provide a good representation of Swiss rural, urban areas and language regions, the results may not be generalizable to all Switzerland. A further limitation is that it is not possible to discriminate between different palliative home care settings, which are influenced by different patient trajectories and can result in different challenges than the ones identified here.
